# A Preliminary Smartphone-Based IMU System for Walking, Running, and Cycling Recognition Under Different Carrying Modes

**DOI:** 10.3390/s26196100

**Published:** 2026-09-26

**Authors:** Longyi Tang, Youli Liang, Binyu Yan

**Affiliations:** 1 College of Software Engineering, Sichuan University, Chengdu 610207, China; tan-glongyi@stu.scu.edu.cn (L.T.); liangyouli@stu.scu.edu.cn (Y.L.); 2 National Key Laboratory of Fundamental Algorithms and Models for Engineering Simulation, Sichuan University, Chengdu 610207, China

**Keywords:** smartphone IMU, motion recognition, on-device inference, carrying mode, accelerometer and gyroscope

## Abstract

This paper presents a preliminary smartphone-based inertial measurement unit (IMU) system for recognizing three common motion types, namely walking, running, and cycling, under different carrying modes. The proposed system is implemented on an Android smartphone and uses accelerometer and gyroscope signals to support both offline evaluation and on-device recognition. The current dataset contains six motion-carrying combinations formed by three motion types and two carrying modes, with more than 15 data segments collected for each category. Each segment lasts approximately 40–60+ s and was collected on flat ground. The raw sensor streams were processed using a sampling rate of 50 Hz, an 8 s sliding window, and a 4 s step size. To reduce data leakage, session-level splitting was adopted in the offline experiments. A K-nearest neighbor (KNN) classifier was used as the baseline recognition model, and different sensor combinations and feature settings were compared. The best offline configuration, using fused accelerometer and gyroscope features with the enhanced feature set and K = 3, achieved an accuracy of 86.57% and a macro-F1 score of 88.42%. On-device tests further showed stable recognition performance under hand-held conditions, while performance decreased under an unseen briefcase-style carrying condition, especially for walking and running. These results indicate that the proposed system is feasible as a preliminary smartphone-based motion recognition pipeline. At the current stage, the study should be interpreted as a pilot-scale feasibility evaluation under limited and partially unbalanced multi-subject data conditions, rather than a strict subject-independent generalization study. The findings nevertheless highlight the importance of carrying-mode diversity and more rigorous cross-subject evaluation in future work.

## 1. Introduction

Smartphone-based motion recognition has attracted increasing attention because modern smartphones are widely available and equipped with inertial sensors, including accelerometers and gyroscopes [1]. These sensors provide an accessible platform for daily activity monitoring, exercise tracking, and context-aware mobile applications. Recent research has investigated daily-life activity and context recognition using smartphone inertial sensors in uncontrolled environments [2]. Stojchevska et al. further explored activities of daily living detection using smartphone data and complementary ambient sensors, illustrating the broader relevance of smartphone sensing to everyday monitoring [3]. These studies motivate continued investigation of practical smartphone-based recognition systems, while also highlighting the diversity of sensing configurations and application settings.

Among common daily and exercise-related motions, walking, running, and cycling are meaningful targets for exercise logging and personal activity monitoring. Recognizing these motion types directly on smartphones can support behavior-aware feedback and lightweight activity analysis. However, reliable recognition in real-world usage remains challenging because sensor readings are influenced by device placement, carrying style, and user interaction patterns. Kundu et al. investigated a CNN-based recognition framework that addresses differences in smartphone configurations and usage behavior [4]. Zhang et al. proposed a smartphone-based activity recognition scheme specifically addressing varying orientations and positions [5]. These studies show that placement and device variability remain active research topics, motivating explicit examination of carrying-condition mismatch in practical smartphone systems.

One important factor in smartphone-based motion recognition is the carrying mode. Previous studies have examined the impact of phone placement on activity recognition and the recognition of locomotion activities with a smartphone in the pocket or hand [6,7]. Related work has also investigated fine-grained carrying states during walking, joint classification of activity and smartphone holding mode, and sensor placement in a minimal smartphone–IMU setup [8,9,10]. Different carrying modes can alter how body motion is transmitted to the device and change the resulting inertial signal characteristics, even when the underlying activity remains the same. Consequently, models trained under a limited set of carrying conditions may perform poorly when the phone is carried differently. Studying such variation is therefore important for developing practical on-device recognition systems.

In this work, we present a preliminary smartphone-based IMU system for recognizing walking, running, and cycling under different carrying modes. The system is implemented on Android and supports both offline analysis and online on-device inference. Accelerometer and gyroscope signals are collected and processed using a fixed sliding-window strategy, and a K-nearest neighbor (KNN) classifier is employed as the baseline recognition model. In the current stage, the study focuses on a pilot-scale dataset collected on flat ground, with six motion-carrying combinations formed by three motion types and two carrying modes. To obtain a more reliable offline evaluation, session-level splitting is adopted instead of random window-level splitting. In addition, on-device tests are conducted to examine the practical behavior of the trained model in real smartphone usage. Accordingly, the present study focuses on an end-to-end feasibility pipeline and practical carrying-mode analysis, rather than proposing a new classifier architecture or an exhaustive multi-model benchmark.

The main contributions of this study are as follows. First, we develop a complete smartphone IMU pipeline that covers data collection, preprocessing, offline model evaluation, and online recognition on an Android device. Second, we provide a preliminary analysis of motion recognition under different carrying modes using accelerometer and gyroscope fusion. Third, we compare different feature settings and sensor combinations and identify a practical baseline configuration for the current dataset. Finally, we report both offline experimental results and on-device observations, including a brief unseen carrying-condition test, and discuss the limitations of the current system as well as possible directions for future improvement.

The remainder of this paper is organized as follows: Section 2 describes the system design, data collection process, preprocessing strategy, feature extraction method, and evaluation setup. Section 3 presents the offline and on-device experimental results. Section 4 discusses the main findings, limitations, and implications of the current study. Section 5 concludes the paper and outlines future work.

## 2. Materials and Methods

### 2.1. System Overview

The proposed system consists of two tightly connected parts: an offline recognition pipeline and an Android-based on-device recognition application. The offline pipeline is used for data preprocessing, feature extraction, model training, and performance evaluation, while the Android application is responsible for continuous sensor collection and online motion recognition. This design allows the same recognition logic to be used in both offline experiments and practical deployment. An overview of the complete pipeline is shown in Figure 1.

During data collection, the smartphone records tri-axial accelerometer and gyroscope signals together with session information. The recorded raw data are then processed offline to generate sliding windows for feature extraction and model training. After the best recognition configuration is selected, the trained model parameters are exported and deployed to the Android application. During online use, the application continuously acquires sensor data, applies the same windowing and feature extraction strategy, and performs on-device prediction using the stored model configuration.

In the current implementation, the recognition target is three-class motion recognition, including walking, running, and cycling. Although the collected data contain two carrying modes, namely hand-held and non-hand-held conditions, the final recognition label is the motion category rather than the carrying mode. In this way, carrying mode is treated as an influencing factor in the evaluation rather than a direct prediction target. This setting is more consistent with practical exercise-monitoring scenarios, where the main purpose is to recognize the user’s current motion regardless of how the smartphone is carried.

The overall system is lightweight and does not rely on cloud inference. All online predictions are produced directly on the smartphone, which makes the system suitable for practical on-device motion recognition with lightweight computation and privacy-preserving deployment. The current study adopts a K-nearest neighbor (KNN) classifier as a practical baseline model, and both baseline and enhanced feature settings are investigated in the offline experiments.

### 2.2. Data Collection

The current dataset was collected using a smartphone-based data acquisition application developed for this study. The data collection focused on three common motion types, namely walking, running, and cycling, and two carrying modes. By combining the motion types and carrying modes, six data categories were obtained in total. For each category, more than 15 data segments were collected, and each segment lasted approximately 40–60+ s. All data were collected on flat ground in order to reduce the influence of slope changes and terrain complexity in this preliminary study. A summary of the current dataset composition is provided in Table 1.

The current data collection was conducted by four participants. Due to practical constraints during the preliminary stage, not all participants covered all six motion-carrying combinations, and the data organization was therefore not fully balanced at the participant level. In addition, the current historical data records did not explicitly preserve a complete subject-to-category mapping for all historical sessions. Accordingly, the present study does not claim a strict subject-independent evaluation. Instead, it is positioned as a preliminary feasibility study focusing on the performance of the proposed smartphone IMU pipeline under the available data conditions. The adopted session-level split reduces overlap-induced leakage between training and testing windows from the same recording session, but it should not be interpreted as equivalent to subject-independent cross-validation.

For each session, the smartphone recorded raw accelerometer and gyroscope streams, and the sampling process also preserved corresponding session-level metadata. In the offline stage, the six original motion-carrying combinations were merged into three motion labels, namely walking, running, and cycling, so that the main experimental task became motion recognition across different carrying conditions. This design reflects the practical objective of recognizing user motion type rather than recognizing the specific carrying configuration itself.

In addition to the offline dataset, supplementary on-device tests were performed after model deployment. These tests were intended to examine practical recognition behavior in real usage conditions rather than to form a fully controlled quantitative carrying-mode benchmark. It should be noted that in part of the on-device validation, a briefcase-style carrying condition was used, whereas the original training data for non-hand-held conditions mainly came from backpack- or pocket-like settings. Therefore, that part of the online test should be regarded as a preliminary out-of-distribution carrying-condition probe rather than an in-distribution validation or a formal multi-carrying-mode comparison.

### 2.3. Preprocessing and Windowing

The raw inertial data were processed using a fixed sampling rate of 50 Hz. A sliding-window strategy was applied to segment the continuous sensor streams into short analysis units for feature extraction and classification. Specifically, the window length was set to 8 s, and the step size was set to 4 s, corresponding to 400 samples per window and 200 samples per step. This setting was consistently used in both offline processing and the Android on-device recognition module. The 8 s window was selected to provide sufficient temporal coverage for capturing relatively stable periodic motion characteristics and frequency-related patterns for walking, running, and cycling under the 50 Hz sampling rate. The 4 s step size was chosen as a practical compromise between temporal overlap, prediction stability, and on-device update frequency. In other words, this setting allows the model to observe enough motion context while still producing refreshed predictions at a usable interval during smartphone-side testing. A more systematic ablation over different window lengths and step sizes will be an important direction for future optimization. Compared with directly using random window-level splitting, the current study adopted a session-level split for offline evaluation. More specifically, all recording sessions were first grouped by the merged motion label, i.e., walking, running, and cycling. Within each motion class, the sessions were randomly shuffled using a fixed random seed of 42, and 80% of the sessions were assigned to the training set, while the remaining 20% were assigned to the test set. When a class contained at least two sessions, the split strategy ensured that both the training set and the test set contained at least one session from that class. All windows generated from the same recording session were kept exclusively in either the training set or the test set so that no overlapping windows from the same original recording could appear in both sets. This protocol provides a more reliable leakage-controlled estimate for the current dataset, but it is still different from a strict subject-independent evaluation because sessions from the same participant may remain within the same side of the split.

Before feature extraction, each window was organized according to the selected sensor mode. Three sensor settings were examined in the offline experiments: accelerometer only, gyroscope only, and accelerometer–gyroscope fusion. The resulting windows were then transformed into feature vectors and normalized using the feature statistics computed from the training data. The same normalization parameters were later reused in the on-device inference stage to ensure consistency between offline training and online deployment.

This preprocessing strategy provides a unified pipeline from raw IMU data to model input. It also makes it possible to compare different feature settings and sensor combinations under the same evaluation framework. In the current study, this consistency is particularly important because the main purpose is not only to achieve reasonable recognition performance offline but also to ensure that the selected model can be directly deployed and tested on the smartphone.

### 2.4. Feature Extraction

For each 8 s window, handcrafted features were extracted from the selected sensor channels: the three accelerometer axes (ax, ay, az), the three gyroscope axes (gx, gy, gz), or all six channels under the fusion setting. In the baseline feature setting, two features were computed for each selected channel: the mean value in the time domain and the dominant frequency obtained from the frequency spectrum. Therefore, the baseline feature dimension was 6 for the accelerometer-only setting, 6 for the gyroscope-only setting, and 12 for the fused accelerometer–gyroscope setting. In the enhanced feature setting, four features were computed for each selected channel: mean, standard deviation, energy, and dominant frequency. Accordingly, the enhanced feature dimension was 12 for either the single-sensor setting or 24 for the fused setting.

The baseline feature set was designed as a simple low-dimensional representation for rapid deployment and initial evaluation. It mainly included statistical and frequency-related descriptors extracted from each sensor axis, such as the mean value and the dominant frequency component. This setting provided a compact baseline for testing whether basic motion patterns could already be separated under the current dataset conditions.

To improve discriminative ability, an enhanced feature set was further introduced. In addition to the baseline descriptors, the enhanced feature set included a richer collection of statistics and signal characteristics derived from accelerometer and gyroscope signals. These descriptors were designed to better capture signal variation, intensity, and motion rhythm within each window. The enhanced setting also incorporated fused information from both sensor modalities, allowing the classifier to use complementary motion cues from linear acceleration and angular velocity.

Three sensor configurations were evaluated in this study: accelerometer only, gyroscope only, and accelerometer–gyroscope fusion. Under the fusion setting, features from both sensors were concatenated into a single feature vector. After feature extraction, min-max normalization was applied based on the training set statistics, and the same normalization parameters were stored and reused during smartphone-side inference. This design ensured that the online recognition module used feature inputs consistent with the offline training stage.

The feature extraction strategy in this work was intentionally kept lightweight to support straightforward implementation and consistent offline and on-device processing. Feature-level fusion has also been investigated in deep learning-based smartphone activity recognition. Raja Sekaran et al. proposed a temporal network that extracts features separately from individual sensor streams before combining them for classification [11]. In contrast, the present study concatenates explicitly defined statistical and frequency-related descriptors and uses a KNN classifier. The two approaches share the general objective of combining information from multiple sensors but differ in how the features are obtained. The current handcrafted representation is used as a preliminary baseline and is not claimed to outperform learned feature representations.

### 2.5. KNN-Based Recognition

A K-nearest neighbor (KNN) classifier was adopted as the baseline recognition model in the current study. This choice was motivated by its straightforward implementation and suitability for establishing a transparent reference pipeline under the available limited-data conditions. KNN also allows the same stored training vectors, normalization parameters, and distance-based decision rule to be reused during on-device inference. However, its prediction cost and storage requirements depend on the number and dimensionality of the retained training vectors. Accordingly, its suitability for the current prototype should not be interpreted as evidence that KNN is universally more resource-efficient than alternative classifiers.

For each test window, the model computes the distance between the normalized feature vector and the stored feature vectors in the training set. The motion label is then determined according to the nearest neighbors under the selected configuration. In the offline experiments, different K values were compared, including the original nearest-neighbor setting and multi-neighbor voting settings. The purpose was to determine whether a slightly more stable local voting mechanism could improve robustness over the simplest one-neighbor decision rule.

The final model selection was based on offline experimental performance under the session-level split protocol. Among the evaluated configurations, the best-performing model used accelerometer–gyroscope fusion, the enhanced feature set, and K = 3. This configuration achieved the highest overall recognition performance and was therefore exported for on-device deployment in the Android application.

It should be noted that the KNN model in this work is used as a practical lightweight baseline rather than a claim of methodological novelty. The main purpose of the present study is to validate the feasibility of the complete smartphone IMU pipeline, from raw data acquisition and leakage-controlled offline evaluation to Android-side periodic on-device deployment under different carrying conditions. Therefore, the emphasis of this work is placed on end-to-end system feasibility and carrying-mode-related behavior rather than on exhaustive classifier benchmarking. A broader comparison with other lightweight models, such as decision trees, random forests, and TinyML-oriented classifiers, will be an important next step in future work.

### 2.6. Offline Evaluation Setup

The offline experiments were designed to compare different sensor configurations, feature settings, and KNN parameters under a unified evaluation pipeline. The main recognition task was three-class motion classification, namely walking, running, and cycling. Although the raw dataset was originally collected under six motion-carrying combinations, the carrying-mode labels were merged at the final classification stage so that the model focused on motion recognition across carrying conditions. Accordingly, the current offline evaluation reports overall three-class motion recognition results across the available carrying conditions, rather than a fully separated carrying-mode-specific benchmark.

The evaluated sensor settings included accelerometer only, gyroscope only, and accelerometer–gyroscope fusion. For each sensor setting, both the baseline feature set and the enhanced feature set were tested. Multiple KNN configurations were then applied to these feature representations. In this way, the experiments aimed to determine how sensor fusion, richer handcrafted features, and neighbor selection affected recognition performance.

The offline evaluation was conducted using a session-level training-test split in order to reduce the influence of overlap-induced leakage between adjacent windows. Performance was measured using accuracy, macro-precision, macro-recall, and macro-F1 score. In addition, confusion matrices were generated for the best configuration to analyze class-specific errors and motion-type confusion patterns. These metrics were selected because the current dataset, while reasonably balanced in terms of class coverage, still requires class-aware evaluation beyond overall accuracy alone. The complete train–test assignment of recording sessions was exported and stored as a split summary file to support reproducibility of the offline experiments.

The offline results were also used to guide the online deployment stage. After all configurations were compared, the best-performing model was exported into a lightweight parameter file for use in the smartphone application. This allowed the same recognition logic, normalization parameters, and label mapping to be shared between offline evaluation and real on-device testing.

### 2.7. On-Device Testing

After the best offline model was selected, the model parameters were exported and deployed to the Android application for smartphone-side testing. The on-device testing was designed to provide a practical validation of whether the offline-selected configuration could maintain reasonable recognition behavior during real usage. Unlike the offline experiments, which were based on recorded datasets and controlled evaluation splits, the online tests focused on direct observation of prediction outputs generated in real time on the smartphone.

During each test, the user started the application and maintained a single motion type under a fixed carrying mode for a short testing period. Multiple short test sessions were conducted for each condition, and the resulting prediction sequences were recorded separately. Therefore, the summary counts were aggregated from multiple short on-device test sessions rather than repeated outputs from one single uninterrupted continuous recording. The smartphone continuously collected accelerometer and gyroscope signals and performed prediction using the same preprocessing and feature extraction strategy as the offline pipeline. Because the online module used an 8 s window and a 4 s step size at 50 Hz, the first prediction became available only after the initial 8 s window was filled, and subsequent predictions were refreshed every 4 s thereafter. To facilitate later inspection without interfering with natural motion, the prediction sequence generated during each test session was stored as a text record together with the raw sensor files. In this study, the briefcase-style condition was not part of the original non-hand-held training data and was therefore treated as an unseen carrying configuration.

The on-device tests covered both hand-held and non-hand-held conditions. In the hand-held setting, the smartphone was directly used in a manner consistent with the trained hand-mode data. In the non-hand-held setting, however, part of the validation used a briefcase-style carrying condition that had not appeared in the original training data, where non-hand-held data mainly came from backpack- or pocket-like conditions. Therefore, the briefcase-style tests should be interpreted as a preliminary unseen carrying-condition probe rather than a standard matched-condition evaluation or a complete quantitative comparison across carrying modes.

The purpose of the on-device testing was not to provide a large-scale benchmark but to examine whether the selected model remained usable after deployment and to identify practical weaknesses that might not be fully visible from offline metrics alone. In particular, these tests were useful for analyzing the impact of carrying-mode mismatch on real-time recognition behavior.

## 3. Results

### 3.1. Offline Experimental Results

The offline experiments compared different combinations of sensor settings, feature sets, and KNN parameters for the three-class recognition task. A summary of the main offline configurations and their recognition results is presented in Table 2.

Here, the reported feature dimension refers to the total number of handcrafted features after concatenating the features extracted from all selected sensor channels.

Overall, the results showed that both sensor fusion and richer handcrafted features substantially improved recognition performance. In contrast, the gyroscope-only setting produced noticeably weaker results, indicating that angular velocity alone was insufficient for stable discrimination among walking, running, and cycling under the current dataset conditions. All reported offline results were obtained under the same session-level split protocol described in Section 2.3.

Among all tested configurations, the best-performing model was the fusion-enhanced KNN model with K = 3. This configuration achieved an accuracy of 86.57%, a macro-precision of 88.55%, a macro-recall of 89.56%, and a macro-F1 score of 88.42%. These results indicate that the combination of accelerometer and gyroscope information, together with the enhanced feature set, provided the most effective representation for the current preliminary recognition task.

A clear performance gap was also observed between the baseline and enhanced feature settings. For example, under the sensor-fusion condition, the enhanced feature set outperformed the baseline feature set by a considerable margin. This result suggests that the richer handcrafted descriptors contributed useful motion-related information beyond the most compact statistical and dominant-frequency features. In other words, lightweight feature engineering remained important even though the classifier itself was relatively simple.

The comparison among sensor configurations further showed a consistent ranking pattern. The fusion setting achieved the best overall performance, the accelerometer-only setting provided intermediate performance, and the gyroscope-only setting produced the weakest results. This pattern indicates that linear acceleration was more informative than angular velocity in the current task, while the fusion of both modalities offered the most robust performance. Therefore, the fusion-enhanced KNN model with K = 3 was selected as the final offline model and deployed to the smartphone application for on-device validation.

### 3.2. Confusion Matrix Analysis

To better understand the behavior of the best offline model, the confusion matrix of the fusion-enhanced KNN configuration with K = 3 was further analyzed. The corresponding confusion matrix is shown in Figure 2.

Overall, the model showed strong discrimination ability for running and relatively stable recognition for walking, while cycling remained the most challenging class under the current dataset conditions.

The running class achieved the clearest separation. Most running windows were correctly recognized as running, and only very limited confusion with the other two classes was observed. This result suggests that running generated more distinctive inertial patterns in terms of rhythm and motion intensity, making it easier for the current handcrafted feature representation and KNN classifier to identify.

Walking also achieved relatively good recognition performance, although a small number of walking windows were misclassified as cycling or running. Compared with running, walking involved milder motion intensity and more overlap with other periodic activities, which may explain the remaining classification errors. Nevertheless, walking was still recognized with acceptable stability in the offline experiments.

Cycling was the most error-prone class in the confusion matrix. A notable portion of cycling windows were misclassified as walking, indicating that the current feature representation did not fully separate these two motion patterns. One possible explanation is that both activities can exhibit relatively regular rhythmic structure under flat-ground conditions, especially when the phone is not rigidly fixed to the body. In such cases, the dominant low-frequency motion pattern and overall signal stability may partially overlap in some windows, while the current lightweight handcrafted features may not capture enough orientation-robust or motion-coupling information to fully distinguish them. In addition, carrying-mode variation may attenuate or distort class-specific movement cues, further reducing separability between cycling and walking. Future improvements may therefore include richer spectral descriptors, energy-distribution features, acceleration–gyroscope coupling features, and other orientation-robust motion descriptors to better reduce this confusion.

Therefore, the confusion analysis indicates that the proposed offline pipeline is already effective for preliminary three-class recognition, but it still has limited robustness in distinguishing cycling from walking. This limitation is important because it reveals that the current system performance is not uniformly strong across all classes. It also suggests that richer data coverage and more expressive lightweight models may be needed in future work, particularly for carrying-mode-sensitive scenarios.

### 3.3. On-Device Test Results

After deployment of the best offline model to the Android application, on-device tests were conducted to observe the practical recognition behavior of the smartphone system. A summary of the on-device test results is provided in Table 3. The results showed a clear difference between matched hand-held conditions and an unseen briefcase-style carrying condition.

Therefore, the briefcase-style results should be interpreted as a preliminary small-scale out-of-distribution carrying-condition test rather than a matched-condition validation or a formal quantitative comparison across carrying modes. Under the hand-held condition, the on-device recognition results were highly stable. These counts summarize prediction outputs aggregated across multiple short test sessions under each condition, rather than repeated predictions obtained from one single continuous recording session. In the current test set, walking, running, and cycling all achieved perfect recognition for the recorded hand-held trials. More specifically, the hand-held tests yielded 19/19 correct predictions for walking, 20/20 for running, and 20/20 for cycling. These observations suggest that the selected offline model can be effectively transferred to the smartphone application when the online usage condition is consistent with the carrying mode represented in training.

In contrast, the recognition performance decreased substantially under the briefcase-style carrying condition. This carrying condition was not used in the original training data, where the non-hand-held samples mainly came from backpack-like or pocket-like settings. Therefore, the briefcase-style evaluation can be interpreted as an unseen carrying-condition test. In this case, walking achieved 9/22 correct predictions, running achieved 9/21, and cycling achieved 20/25. The corresponding recognition rates were 40.9%, 42.9%, and 80.0%, respectively.

A closer inspection showed that walking and running were particularly sensitive to this carrying-mode mismatch. Their prediction sequences became less stable, and the model more frequently confused them with other classes. Cycling remained more robust than the other two classes even under the unseen briefcase-style condition, although its performance also declined compared with the hand-held setting. These results indicate that carrying-mode shift has a substantial impact on real-world motion recognition performance, especially when the new carrying condition has not been represented during training.

Taken together, the on-device results support two conclusions. First, the proposed smartphone IMU pipeline is practically usable under matched hand-held conditions, where offline and online behaviors are highly consistent. Second, the generalization ability of the current model is limited under unseen carrying configurations. This finding highlights the importance of collecting more diverse carrying-mode data and incorporating broader real-world variation in future work.

## 4. Discussion

The results of this study show that a lightweight smartphone-based IMU pipeline can provide effective preliminary recognition of walking, running, and cycling, especially when the online carrying condition is consistent with the training setting. The offline experiments demonstrated that sensor fusion and enhanced handcrafted features substantially improved recognition performance over simpler baseline settings. In particular, the fusion-enhanced KNN configuration with K =3 achieved the best overall balance among accuracy, macro-precision, macro-recall, and macro-F1 score, indicating that even a relatively simple classifier can provide useful performance when the preprocessing and feature design are properly configured.

Another important finding is the clear difference between matched and unseen carrying conditions in on-device testing. Under the hand-held condition, the deployed model showed stable recognition behavior in the recorded trials. However, under the briefcase-style carrying condition that was not included in the training data, performance dropped noticeably, especially for walking and running. These observations are consistent with the broader concern about placement-dependent signal variation examined in earlier placement and locomotion studies [6,7,10] and in more recent work addressing device usage behavior and varying phone orientations and positions [4,5]. Nevertheless, the current small-scale tests do not establish the effectiveness of any position-independent recognition strategy. A more complete evaluation across pocket, backpack, waist-mounted, and briefcase-style placements, with controlled participant coverage and repeated sessions, remains an important next step.

The confusion analysis also provides useful insight into the current limitations of the system. Running was relatively easy to recognize, which is likely related to its stronger rhythmic and intensity-related inertial patterns. By contrast, cycling showed more frequent confusion with walking in the offline experiments. A likely reason is that both activities can present partially overlapping periodic structure and relatively smooth low-frequency motion patterns in some windows, particularly under flat-ground conditions and variable carrying modes. Under these conditions, the present lightweight handcrafted features may not fully preserve the motion-specific differences needed for consistent separation. This finding suggests that future work should consider richer spectral descriptors, energy-distribution features, orientation-robust representations, and acceleration–gyroscope coupling features to improve discrimination between cycling and walking.

Several limitations of the current study should be noted. First, the dataset size is still limited, and the participant-to-category organization was not fully balanced. Second, the current historical data records do not completely preserve a clean subject-level mapping for all sessions, which means that a rigorous subject-independent evaluation was not established at this stage. Therefore, although the adopted session-level split helps reduce overlap-induced leakage, the current offline results should not be interpreted as strong evidence of cross-subject generalization. Third, the current study only considered flat-ground scenarios and did not include more complex environments such as slopes, stairs, or rough outdoor surfaces. Accordingly, the present work should be interpreted as a preliminary feasibility study rather than a complete large-scale benchmark. 

Future work should prioritize balanced multi-subject data collection and broader carrying-condition coverage. Methodological extensions could include representations addressing device and placement variability [4,5] and learned feature-level sensor fusion [11]. Sangisetti and Pabboju investigated an enhanced CNN for activity recognition using the MHealth benchmark [12]. However, that benchmark uses a different sensing setup from the smartphone-only system studied here, so its reported performance should not be treated as a directly comparable baseline. Future model comparisons should use a common evaluation protocol and consider recognition performance, storage requirements, inference time, and energy consumption.

## 5. Conclusions

This paper presented a preliminary smartphone-based IMU system for recognizing walking, running, and cycling under different carrying modes. The proposed framework integrates offline preprocessing and evaluation with online Android-based inference, thereby forming a complete end-to-end motion recognition pipeline. Using accelerometer and gyroscope fusion together with an enhanced handcrafted feature set, the best KNN configuration achieved an offline accuracy of 86.57% and a macro-F1 score of 88.42%.

The on-device tests further showed that the trained model could achieve stable recognition under hand-held conditions, while performance decreased under an unseen briefcase-style carrying condition. These results indicate that the proposed system is feasible for practical smartphone-based motion recognition but also demonstrate that carrying-mode diversity remains a critical factor for generalization.

Overall, the present work provides a useful baseline for future research on lightweight on-device motion recognition. Future improvements will focus on expanding the dataset, improving carrying-mode coverage, introducing more systematic subject-level evaluation, and exploring lightweight deep learning methods for stronger robustness in real-world smartphone usage scenarios.

## Figures and Tables

**Figure 1:**
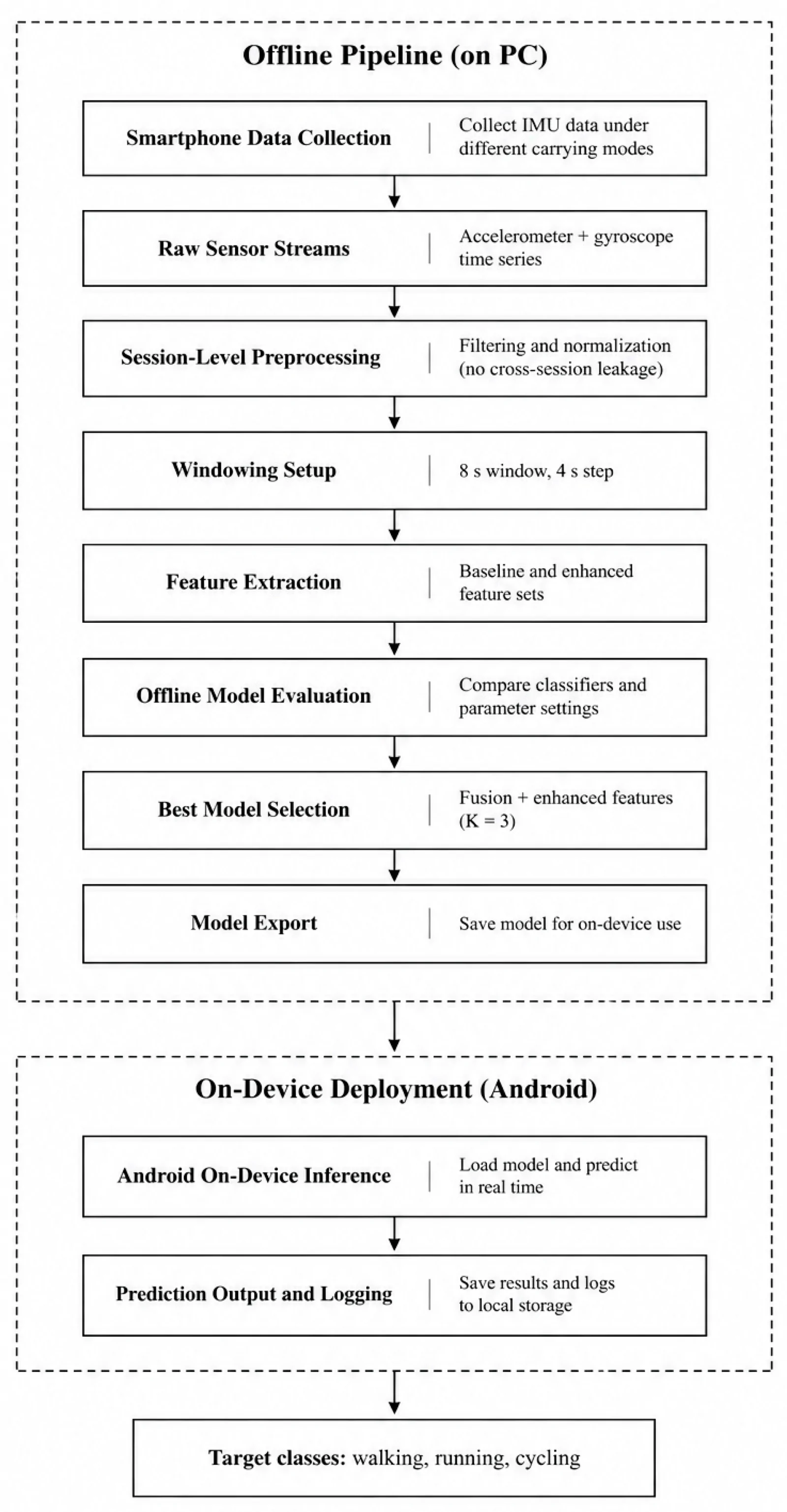
Overview of the proposed smartphone IMU motion recognition pipeline, including data collection, preprocessing, offline training, model export, and on-device inference.

**Figure 2:**
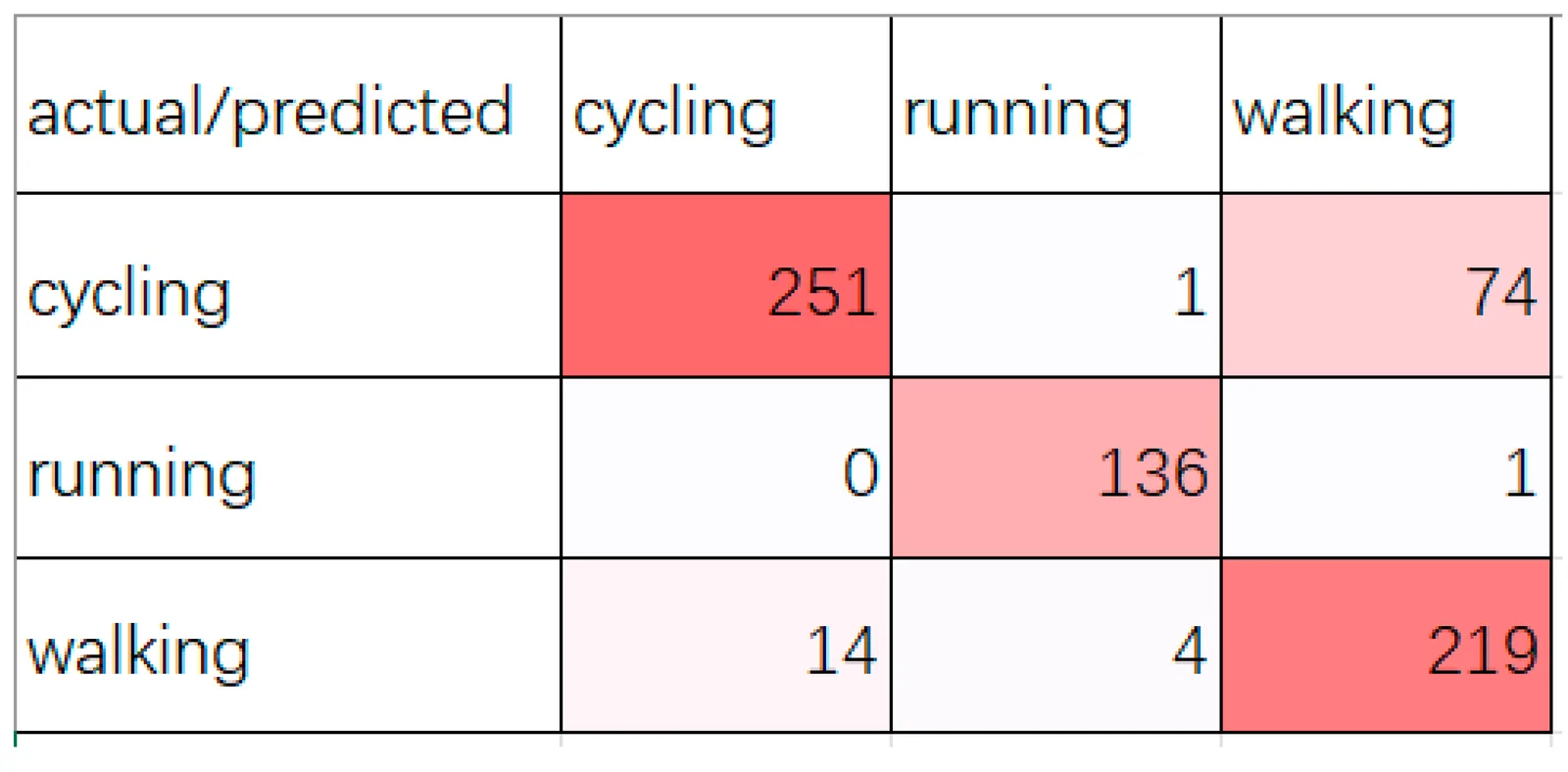
Confusion matrix of the best offline model (fusion-enhanced KNN with K = 3) for three-class motion recognition. Rows represent the actual classes, and columns represent the predicted classes. Cell values indicate the numbers of test windows, with darker red shading indicating larger counts.

**Table 1:** Summary of the current dataset used in this study.

Category	Motion Type	Carrying Mode	Number of Segments	Approximate Duration per Segment (s)	Notes
walking_hand	Walking	Hand-held	15+	40–60+	Flat-ground collection
walking_nonhand	Walking	Non-hand-held	15+	40–60+	Backpack-/pocket-like training condition
running_hand	Running	Hand-held	15+	40–60+	Flat-ground collection
running_nonhand	Running	Non-hand-held	15+	40–60+	Backpack-/pocket-like training condition
cycling_hand	Cycling	Hand-held	15+	40–60+	Flat-ground collection
cycling_nonhand	Cycling	Non-hand-held	15+	40–60+	Backpack-/pocket-like training condition

**Table 2:** Offline recognition results under different sensor and feature configurations.

Experiment ID	Sensor Mode	Feature Set	K	Feature Dimension	Train Windows	Test Windows	Accuracy	Macro Precision	Macro Recall	Macro F1	Cycling F1	Running F1	Walking F1
fusion_baseline_knn1	Fusion	Baseline	1	12	2215	700	0.7786	0.7846	0.8046	0.7928	0.7851	0.8858	0.7073
fusion_baseline_knn3	Fusion	Baseline	3	12	2215	700	0.7943	0.8015	0.8251	0.8097	0.7881	0.9003	0.7406
fusion_baseline_knn5	Fusion	Baseline	5	12	2215	700	0.7914	0.7961	0.8243	0.8062	0.7881	0.8986	0.7320
acc_baseline_knn3	Accelerometer only	Baseline	3	6	2215	700	0.7529	0.7594	0.7839	0.7700	0.7551	0.8912	0.6638
gyro_baseline_knn3	Gyroscope only	Baseline	3	6	2215	700	0.5829	0.5797	0.6168	0.5869	0.6135	0.6410	0.5062
fusion_enhanced_knn3	Fusion	Enhanced	3	24	2215	700	0.8657	0.8855	0.8956	0.8842	0.8494	0.9784	0.8249

**Table 3:** Summary of the supplementary on-device test results under matched and unseen carrying conditions.

Condition	Motion Type	Correct/Total	Recognition Rate (%)	Remarks
Hand-held	Walking	19/19	100.0	Matched condition
Hand-held	Running	20/20	100.0	Matched condition
Hand-held	Cycling	20/20	100.0	Matched condition
Briefcase-style carrying	Walking	9/22	40.9	Unseen carrying condition
Briefcase-style carrying	Running	9/21	42.9	Unseen carrying condition
Briefcase-style carrying	Cycling	20/25	80.0	Unseen carrying condition

## Data Availability

The data supporting the findings of this study are available from the corresponding author upon reasonable request.
